# Phosphoproteomics profiling reveals a kinase network conferring acute myeloid leukaemia intrinsic chemoresistance and indicates HMGA1 phosphorylation as a potential influencer

**DOI:** 10.1002/ctm2.749

**Published:** 2022-03-16

**Authors:** Yinghui Zhu, Xin He, Shu Li, Yichao Gan, Zheng Li, Hanying Wang, Haojie Dong, Lei Zhang, Sheng‐Li Xue, Yang Xu, Ling Li

**Affiliations:** ^1^ Department of Hematological Malignancies Translational Science, Gehr Family Center for Leukemia Research Hematologic Malignancies and Stem Cell Transplantation Institute, Beckman Research Institute, City of Hope Medical Center Duarte California USA; ^2^ Department of Hematology The Second Affiliated Hospital, Zhejiang University School of Medicine Hangzhou China; ^3^ Institute of Genetics, Zhejiang University Hangzhou China; ^4^ Department of Genetics Zhejiang University School of Medicine Hangzhou China; ^5^ Department of Hematology, The First Affiliated Hospital of Soochow University, Jiangsu Institute of Hematology National Clinical Research Center for Hematologic Diseases Suzhou China; ^6^ Institute of Blood and Marrow Transplantation, Collaborative Innovation Center of Hematology Soochow University Suzhou China; ^7^ Zhejiang Provincial Key Laboratory for Cancer Molecular Cell Biology Life Sciences Institute, Zhejiang University Hangzhou China

Dear Editor,

The underlying mechanisms of cancer intrinsic drug resistance^1^ remain elusive. Herein, we report findings relevant to phosphoproteomics of acute myeloid leukaemia (AML) specimens. Specifically, we profiled phosphoproteins of cells from AML patients undergoing chemo‐failure compared with those achieving remission, and identify signatures associated with AML refractoriness.

We collected bone marrow specimens at initial diagnosis from patients with comparable clinical characteristics (Tables S1 and S2); they exhibited either treatment failure (F) or reached complete remission (R) following ‘7 + 3’ induction therapy. We performed quantitative phosphoproteomics and total proteomics (Figure 1A), and found 9181 phosphorylation sites corresponding to 3001 phosphoproteins from phosphor‐proteome, 4648 proteins from total‐proteome (Figure 1B). Due to phosphopeptide distribution and technical consideration (Figure 1C), we focused on phosphor‐serine/threonine. We confirmed high accuracy of the analysis (mass‐errors/peptide length) (Figure S1A‐C) and observed good reproducibility of phosphorylated and total peptides (Figure 1D; Figure S1D). A total of 20% (630) of 3001 phosphoproteins (of which 627 were up‐regulated) and 3% (146) of the 4648 proteins were differentially seen in F relative to the R group (Figure 1E,F). Differential proteins/phosphoprotein criteria was applied as fold‐change [F/R] > 1.5 or < 0.67 and *p *< 0.05). Unsupervised hierarchical clustering analysis to separate failure from remission samples indicated that chemo‐failure cases exhibited a distinct phosphoprotein signature (Figure 1G).

**FIGURE 1:**
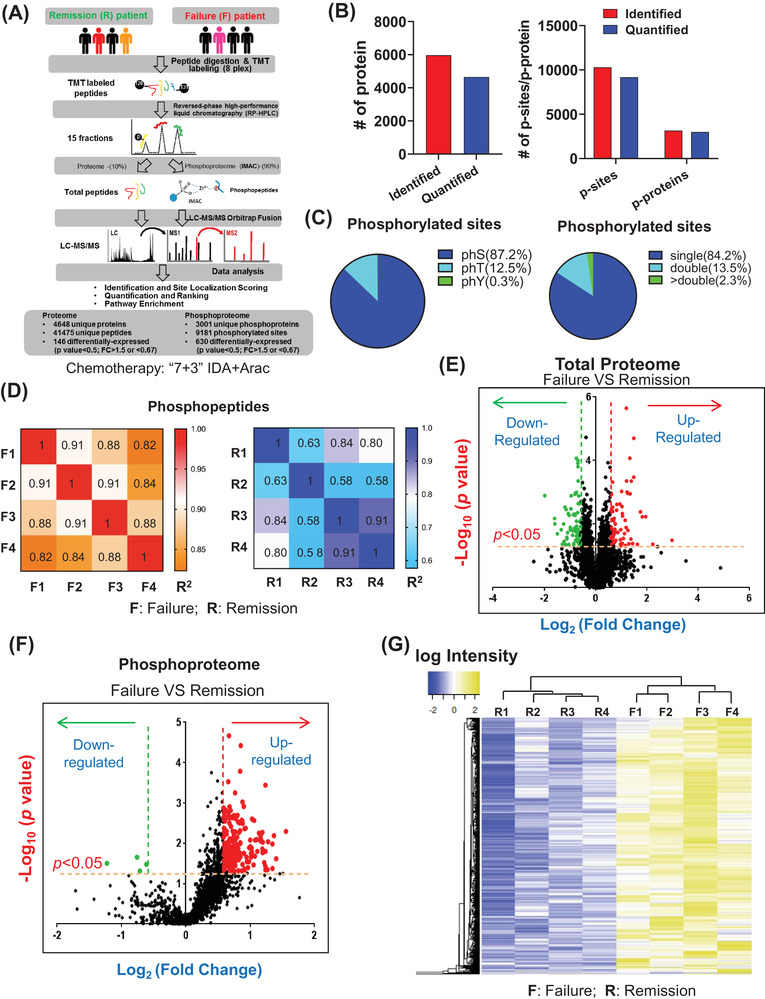
In‐depth phosphoproteomics analysis of chemo‐refractory versus chemo‐sensitive acute myeloid leukaemia (AML) specimens. (**A**) Workflow of proteomics analysis of leukaemia cells from AML patient bone marrow specimens. Cases either exhibited failure (F) during 7 + 3 induction chemotherapy or reached remission (R) after induction chemotherapy (four specimens per group). After digestion, peptides were labelled with TMT reagent and enriched using metal affinity chromatography (IMAC). (**B**) Proteins, phosphosites and phosphoproteins identified and quantified in bone marrow of AML patients. (**C**) Distribution of serine‐ (S), threonine‐ (T) and tyrosine‐ (Y) phosphorylated sites in bone marrow cells from eight AML patients. Distribution of phosphopeptides with one, two or more phosphorylated sites. (**D**) Heatmap showing Pearson's correlation coefficients (*R* ^2^) of phospho‐proteome data, indicating reproducibility among individual patient‐derived samples, among failure or remission groups. (**E**) Volcano plot displaying distribution of proteins with relative protein abundance (*x*‐axis: Log_2_ fold‐change;cut‐off: 0.5849625 or −0.57767; linear fold‐change (failure [F]/remission[R] > 1.5 or < 0.67) plotted against significance level (*y*‐axis:–Log_10_ *p*‐value;cut‐off:1.30103, linear *p*‐value < 0.05), showing significantly (*p *< 0.05) increased (failure/remission >1.5, in red) or decreased (failure/remission < 0.67; green) phosphorylated proteins in chemo‐failure patients. (**F**) Volcano plot displaying the distribution of phosphorylated proteins with relative protein abundance (*x*‐axis: Log_2_ fold‐change; cut‐off: .05849625 or −0.57767; linear fold‐change (failure [F]/remission[R] > 1.5 or < 0.67) plotted against significance level (*y*‐axis:–Log_10_ *p*‐value;cut‐off:1.30103, linear *p*‐value < 0.05), showing significantly increased (failure/remission >1.5; red) or decreased (failure/remission <0.67; green) phosphorylated proteins in chemo‐failure patients. (**G**) Heatmap showing an abundance of 630 differentially phosphor‐proteins after unsupervised hierarchical clustering in eight individual samples. Results indicate separation between failure and remission groups

Ingenuity pathway analysis (IPA) identified that DNA damage response pathway was top‐ranked (Figure 2A). ATM signalling was up‐regulated in chemo‐failure specimens, consistent with others.^2^ FLT3, ERK/MAPK and Rho kinase signalling pathways were also enriched in F samples, as reported^3^ (Figure S2A). We next focused on up‐regulated phosphoproteins (627) to identify corresponding kinases. NetworKIN analysis showed that in F group, 250 up‐regulated phosphor‐proteins (confidence score > 2)^4^ are potentially phosphorylated by 54 kinases. Those 250 substrates were more abundant than the remaining phosphoproteins, as evidenced by a shift in intensity (Figure S2B). We further analysed the top 24 kinases (Table S3), of which each was responsible for >1% of total phosphorylation sites (Figure 2B). Those top 24 kinases all function in cell cycle regulation (Figure S2C). In addition to NetworKIN, KEA2 analysis revealed that activity of Casein Kinase II (CK2, gene name: CSNK2A1) or CDK family members was higher in F relative to R specimens (Figure 2C; Table S4). Moreover, DEPMAP analysis revealed that depleting CDK family members in AML cell lines decreased cell viability (a gene with a score < –1 indicates an essential gene) (Figure 2D). Not only identified as a top kinase from NetworKIN analysis (Table S3), CK2 was also the most enriched upstream kinase in chemo‐failure samples through our IPA analysis (Figure 2E). Accordingly, CK2 inhibition by CX‐4945 significantly enhanced cytarabine‐induced cell death in chemo‐failure AML cells (Figure 2F). Thus, we next focused on CK2 substrates in context of haematopoiesis.

**FIGURE 2:**
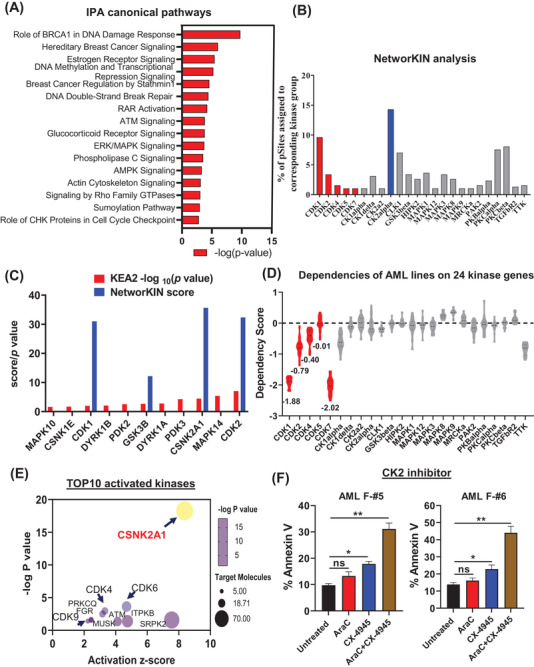
Network analysis reveals kinase signature in refractory acute myeloid leukaemia (AML) specimens. (**A**) The top 15 canonical pathways derived from IPA gene ontology algorithms for 630 differentially phosphorylated proteins. Pathways emerged following IPA ‘Core Analysis’. (**B**) Kinases predicted to be enriched in refractory AML specimens, based on NetworKIN analysis. *Y*‐axis: percentage of phosphor‐sites catalysed by corresponding kinases (384 phosphorylation sites for 250 phosphor‐proteins). (**C**) Significantly activated kinases in chemo‐failure AML specimens based on KEA2 analysis (red bars); blue bars indicate high confidence kinases enriched in failure patients, based on NetworKIN analysis. (**D**) Graph showing effects following knockout of indicated activated kinase in AML cell lines (*n* = 20) (data sourced from the Cancer Dependency Map (https://depmap.org/portal/)). For gene effects, a score <–0.5 represents modest depletion in most cell lines, and a score <−1 represents an essential gene. The dependency score of CDK family members is indicated in red. (**E**) Predicted top 10 activated kinases upon IPA analysis of differentially phosphorylated proteins. (**F**) Apoptosis analysis after 48 h of combination treatment with CX‐4945 (CK2 inhibitor; 5 μM) and/or AraC (2 μM) of failure AML specimens. The apoptotic percentage is indicated by annexin V positivity. Error bars represent standard error of the mean. *p < 0.05, **p < 0.01, ***p < 0.001, ****p < 0.0001

We observed an overlap of 27 differentially phosphorylated proteins between the top two IPA terms relevant to haematopoiesis (Figure 3A; Table S5). We then analysed the top 10 most abundant proteins of the 27 (Figure 3B); among them, KIT, BCR, LAIR1 and RB1 are leukemic oncoproteins.^5^ Interestingly, modified forms of HMGA1 phosphorylated at S99, S102 or S103 were among the top most abundant phosphoproteins in chemo‐failure AML samples (Figure 3C; Table S6). HMGA1 S99/102/103 is highly conserved across species (Figure 3D). Our mass spectrometry analysis in another set of samples verified HMGA1 hyperphosphorylation in chemo‐failure AML specimens (Figure 3E,F; Table S7). Although HMGA1 function in leukemogenesis has been unclear, phosphorylation of HMGA1 S102 is reportedly catalysed by CK2.^6^ We verify the activity by treating primary AML cells with CK2 inhibitor (Figure S3A). It is also noteworthy that as a central hub of the CK2‐substrate network (Figure 3G), HMGA1 interacts with other proteins, such as SP1, which is a critical transcription factor responsible for aberrant expression of many genes which regulate cancer progression.^7^

**FIGURE 3:**
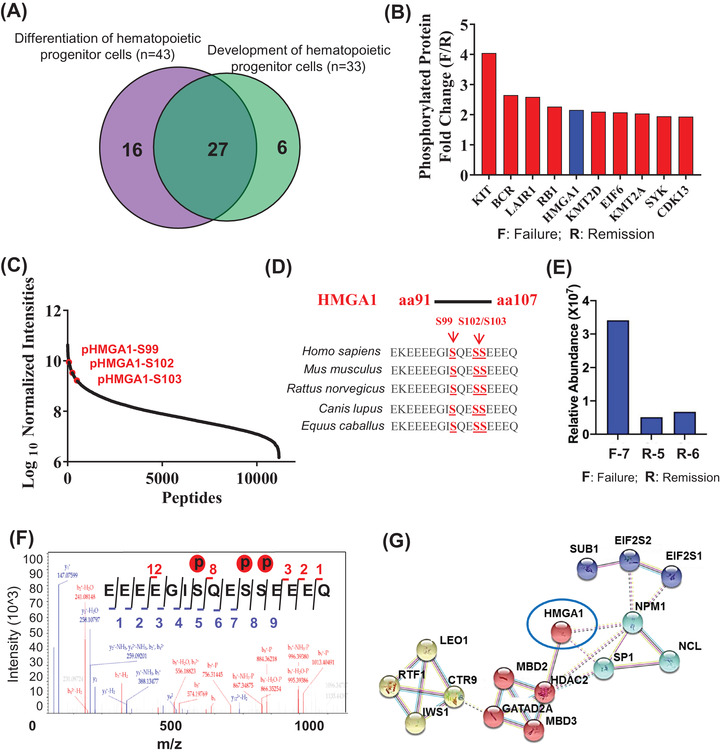
Phosphorylation of HMGA1, a canonical CK2 substrate, is highly expressed in chemo‐failure AML. (**A**) Ingenuity pathway analysis (IPA) analysis of differentially phosphorylated proteins relevant to hematopoietic disease, showing 27 phosphoproteins common in the top two IPA terms associated with haematopoiesis. (**B**) The top 10 abundant differentially phosphoproteins derived from 27 proteins are shown in Figure 3A, the rank is based on fold‐change. (**C**) Phospho‐proteomics screen for all the protein phosphorylation sites in eight AML specimens; pHMGA1 S99, S102 and S103 are marked in red. (**D**) Sequence alignment of HMGA1 proteins from different species showing conserved S99/102/103 sites. (**E**) Relative abundance of phosphorylated HMGA1 among F and R AML patients. (**F**) Representative spectra of phosphorylated HMGA1 peptides including all three p‐sites (S99, S102 and S103). (**G**) Prediction of HMGA1‐interacting proteins based on IPA analysis

IPA analysis suggested that HMGA1 phosphorylation may promote cell survival (Figure S3B). To test this, we used shRNA to knockdown (KD) HMGA1 in AML lines and observed markedly decreased cell growth (Figure 4A; Figure S4A) and induction of G0/G1 cell cycle arrest (Figure S4B,C). We also observed remarkable loss of phosphor‐serine signals after mutating HMGA1 residues S99/S102/S103 (to phosphorylation‐deficient S3A), confirming that they are the primary HMGA1 phosphor‐residues (Figure 4B). We then ectopically expressed HMGA1 constructs mimicking constitutive phosphorylation (S3D) or phospho‐deficiency (S3A) form in *MLL‐AF9*/FLT3‐ITD (MA9/ITD) murine bone marrow cells to assess AML growth regulation (Figure S4D). Enforced S3D expression enhanced CFC of MA9/ITD, while expression of S3A in cells did not (Figure 4C; Figure S4E). Furthermore, serum‐starved S3D‐expressing cells showed enhanced survival relative to similarly treated MOCK cells, while S3A overexpression conferred no survival advantage (Figure 4D). Finally, when we treated MA9/ITD cells with cytarabine (AraC), S3D cells showed some resistance relative to cells expressing S3A or MOCK (Figure 4E‐G).

**FIGURE 4:**
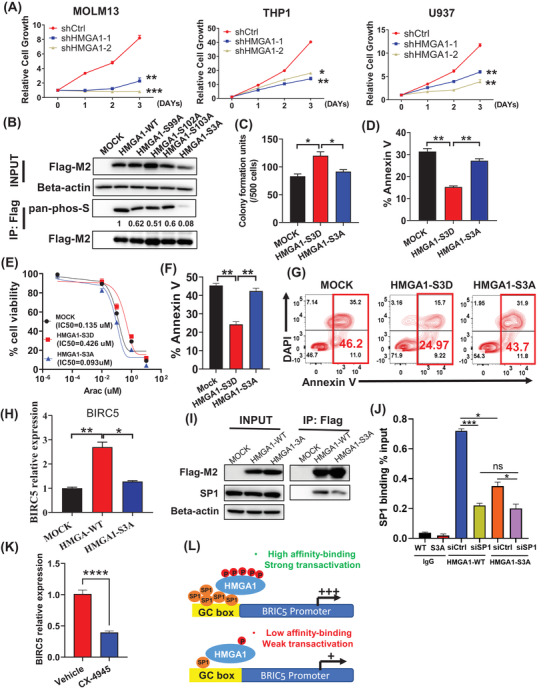
HMGA1 phosphorylation modulates acute myeloid leukaemia (AML) cell chemo‐sensitivity via regulating BIRC5 expression. (**A**) Cell viability analysis of indicated cells transduced with shCtrl or shHMGA1. (**B**) Western blotting for pan‐phospho‐serine levels in 293T cells transduced with MOCK, Flag‐tagged HMGA1‐WT, HMGA1‐S99A, HMGA1‐S102A, HMGA1‐S103A or HMGA1‐S3A. Respective proteins in lysates were pulled down with an anti‐Flag‐M2 antibody. (**C**) Colony‐forming cell assay performed in MA9/ITD cells transfected with mock, HMGA1‐S3D, or HMGA1‐S3A. Note that 500 cells were plated per well. Error bars represent SD from three independent experiments. (**D**) Analysis of apoptosis based on annexin V staining in serum‐starved MA9/ITD cells transduced with indicated constructs. (**E**) Cytotoxicity assays in the presence of varying concentrations of AraC in MA9/ITD cells expressing the indicated constructs. (**F–G**) Apoptosis assay of MA9/ITD cells expressing the indicated constructs after 48 h of 0.25 μM AraC treatment. (**H**) BIRC5 mRNA expression in MOCK, HMGA1‐WT, or HMGA1‐S3A transduced 293T cells. Error bars represent SD from three independent experiments. (**I**) Immunoblotting of Flag‐M2, SP1 and β‐actin in 293T cells transduced with indicated constructs following IP with Flag‐M2 antibody. (**J**) ChIP‐qPCR assay in 293T cells that ectopically overexpress Flag‐tagged HMGA1‐WT or HMGA1‐S3A, and with siSP1 in these Flag‐HMGA1 expressing cells. ChIP analysis using anti‐flag antibodies or control normal mouse IgG was performed in four groups: HMGA1‐WT/siCtrl, HMGA1‐WT/siSP1, HMGA1‐S3A/siCtrl and HMGA1‐S3A/siSP1. Primers used for amplification of a targeted region of the BIRC5 promoter contain some SP1 binding sites (see Supporting Information). Error bars represent SD from three independent experiments. (**K**) Real‐time PCR was performed using BIRC5 taqman‐probes in AML primary cells treated 48 h with the CK2 inhibitor CX‐4945 (5 μM). (**L**) Schema showing the proposed model of HMGA1/SP1/BIRC5 axis. Error bars represent the standard error of the mean. *p < 0.05, **p < 0.01, ***p < 0.001, ****p < 0.0001

HMGA1 is a chromatin‐binding protein that interacts with SP1 to enhance its trans‐activity.^7^ SP1 up‐regulates the expression of BIRC5, an AML relevant anti‐apoptotic gene.^8^ Interestingly, BIRC5 expression was upregulated in 293T cells upon WT HMGA1 overexpression, while S3A overexpression did not have a comparable effect (Figure 4H). We thus asked whether HMGA1 phosphorylation at S99/102/103 promoted SP1 binding to BIRC5 promoter region and increased BIRC5 transcription. Co‐IP analysis demonstrated that mutation of S99/S102/S103 but not threonine 53 (T53)^9^ robustly attenuated HMGA1 binding to SP1 (Figure 4I; Figure S4F). ChIP analysis also revealed that mutating HMGA1 S99/S102/S103 attenuated HMGA1 binding to the BIRC5 promoter; SP1 KD significantly decreased HMGA1 binding to the BIRC5 promoter (*p *< 0.001), but only modestly affected HMGA1‐S3A protein binding to the same region (*p *= 0.0342) (Figure 4J; Figure S4G,H). These suggest that phosphor‐HMGA1 regulation of BIRC5 expression is SP1 dependent. Furthermore, treatment of primary AML cells with CK2 inhibitor not only downregulated HMGA1 phosphorylation levels (Figure S3A) but also decreased BIRC5 levels (Figure 4K). These results support a model that hyper‐phosphorylated HMGA1 enhances SP1 to transactivate BIRC5 (Figure 4L).

Overall, we reveal that HMGA1 phosphorylation promotes intrinsic resistance and that blocking CK2‐mediated HMGA1 phosphorylation may enhance cytarabine‐based chemo‐therapy. We also reveal a kinase signature predictive of chemoresistance and illustrate the importance of proteomics technology for better understanding cancer resistance.

## CONFLICT OF INTEREST

The authors declare no conflict of interest.

## Supporting information

Supporting Information

## References

[1] Zhan JG, Gu Y , Chen B . Mechanisms of drug resistance in acute myeloid leukemia. Onco Targets Ther. 2019; 11(12): 1937–1945. 10.2147/OTT.S191621 PMC6417008 30881045

[2] Blackfor AN , Jackson SP . ATM, ATR, and DNA‐PK: the trinity at the heart of the DNA damage response. Mol Cell. 2017; 66(6): 801–817. 28622525 10.1016/j.molcel.2017.05.015

[3] Azzam DJ , Tay JWT , Greeve MA , Harvey JM , Bental JM . ERK/MAPK regulation of the androgen responsiveness of breast cancer cells. Adv Exp Med Biol. 2008; 617: 429–435. 18497066 10.1007/978-0-387-69080-3_41

[4] Lindin R , Jensen LJ , Pasculescu A , et al. NetworKIN: a resource for exploring cellular phosphorylation networks. Nucleic Acids Res. 2008; 36: D695–D699. 17981841 10.1093/nar/gkm902 PMC2238868

[5] Perbellin O , Falisi E , Giaretta I , et al. Clinical significance of LAIR1 (CD305) as assessed by flow cytometry in a prospective series of patients with chronic lymphocytic leukemia. Haematologica. 2014; 99(5): 881–887. 24415628 10.3324/haematol.2013.096362 PMC4008102

[6] Wan Y‐T , Pan S‐H , Tsai C‐F , et al. Phosphoproteomics reveals HMGA1, a CK2 substrate, as a drug‐resistant target in non‐small cell lung cancer. Sci Rep. 2017; 7: 44021. 28290473 10.1038/srep44021 PMC5349541

[7] Aiell A , Pandini G , Sarfstein R , et al. HMGA1 protein is a positive regulator of the insulin‐like growth factor‐I receptor gene. Eur J Cancer. 2010; 46(10): 1919–1926. 20335021 10.1016/j.ejca.2010.02.050

[8] Mityae M V, Kopantzev EP , Buzdin AA , Vinogradova TV , Sverdlov ED . Functional significance of a putative sp1 transcription factor binding site in the survivin gene promoter. Biochemistry (Moscow). 2008; 73(11): 1183–1191. 19120021 10.1134/s0006297908110035

[9] Zhan Q , Wang Y . Homeodomain‐interacting protein kinase‐2 (HIPK2) phosphorylates HMGA1a at Ser‐35, Thr‐52, and Thr‐77 and modulates Its DNA binding affinity. J Proteome Res. 2007; 6(12): 4711–4719. 17960875 10.1021/pr700571d PMC2547408

