# Supplementary material for: Phosphoproteomics profiling reveals a kinase network conferring acute myeloid leukaemia intrinsic chemoresistance and indicates HMGA1 phosphorylation as a potential influencer
Source: Clin Transl Med. 2022 Mar 16;12(3):e749. doi: 10.1002/ctm2.749 (PMC8926901; doi:10.1002/ctm2.749)
Supplement: Supplementary file 1 — Supporting Information [file CTM2-12-e749-s001.docx]

**Phosphoproteomics Profiling Reveals a Kinase Network Conferring AML Intrinsic Chemoresistance and Indicates HMGA1 Phosphorylation as a Potential Influencer**

**SUPPORTING INFORMATION**

**Materials and Methods**

**Tissue Samples**

Sample acquisition was approved by the COH Institutional Review Board or Soochow University in accordance with the Helsinki Declaration. Detailed information relevant to Failure (F) or Remission (R) is provided in Supplementary Table S1.

**Sample preparation for total proteome and phosphoproteome analysis**

Samples were prepared as described^[1]^, with minor modifications. Briefly, purified mononuclear cells (>80% AML blasts) were lysed in lysis buffer (8M urea, 4% HEPES) supplemented with 1% protease inhibitor and 1% phosphatase inhibitor. Protein concentration was determined by BCA assays. Lysates were reduced with 5 mM DTT, alkylated with 10 mM IAA, and then diluted with TEAB to ensure a urea concentration <2M. Samples were trypsin-digested overnight at a 1:50 ratio of protease to lysate proteins. TMT11-plex reagents were reconstituted according to the manufacturer’s instructions (ThermoFisher Scientific). Tryptic peptides were purified by solid phase extraction, and purified peptides labeled using TMT reagents. To exclude bias due to differential abundance of unmodified proteins, we also performed total proteomic analysis for all specimens. For that analysis, we extracted 500 μg protein from each sample and used 10% for total proteome profiling and the rest for enrichment of phosphorylated peptides. We then subjected samples to liquid chromatography-tandem mass spectrometry (LC-MS/MS) analysis. Phosphopeptides were enriched using immobilized metal affinity chromatography (IMAC) and separated using RP-RP chromatography coupled to a nanoelectrospray ion source.

**Mass spectrometry and data analysis**

Proteomic data were collected on an Orbitrap Fusion mass spectrometer (ThermoFisher Scientific) coupled to a Proxeon EASY-nLC 1000 liquid chromatography (LC) pump (ThermoFisher Scientific). Peptides were separated in 0.1% Formic Acid/2% ACN (buffer A) and 0.1% Formic Acid/90% ACN (buffer B). The flow rate was 300 nL/min on the Easy nLC1000 system. MS1 data were collected using the Orbitrap mass analyzer (120,00 resolution at 200 m/z; 350-1400 m/z; maximum injection time 50 ms; AGC 4e5). Determined charge states between 2 and 5 were required for sequencing, and a 120 s dynamic exclusion window was used. Raw data were processed with Mascot 2.3 (Matrix Science, Boston, MA). An automatic decoy database search was performed. Several parameters in Mascot were set for peptide searching, including TMT11-plex for quantification, tolerance of two missed trypsin cleavage sites, and a maximum peptide charge of five. The minimum peptide length was seven. Also, methylthio for cysteine as a fixed modification, and oxidation for methionine as a variable modification. Following addition of Phospho (ST) as a variable modification for phosphoproteomics studies, the precursor mass tolerance was 10 ppm, and the MS/MS product ion tolerance was 0.02 Da. Unique proteins with at least two unique peptides and a false discovery rate (FDR) < 0.01 were qualified (for further quantification analysis. Fold-changes in protein abundance were defined as the median ratio of all significantly matched spectra with tag signals. The following parameters were used to define differential proteins/phosphoproteins: up-regulated proteins (F AML/R AML fold-change > 1.5, log2 fold-change >0.584963; *p*<0.05); down-regulated proteins (F AML/R AML fold-change <0.67, log 2 fold-change <-0.57777; *p*<0.05). Mass spectrometry data were deposited to the ProteomeXchange Consortium via the PRIDE partner repository. The dataset identifier is PXD030158.

**DNA constructs**

PCDH-EF1A-T2A-GFP/Puro vectors were purchased from System Biosciences, Inc. Full-length HMGA1 mutants including S99A, S102A, S103A, S99/102/103A, and S99/102/103D were cloned into PCDH-EF1A-T2A-GFP, as described ^[2]^. We also used lentiviral shRNA vectors (PLKO.1) targeting HMGA1 (TRCN0000018949 and TRCN0000018951, designated shHMGA1-1 and shHMGA1-2, respectively) obtained from Mission Sigma. shScramble sequence: GCGCGCTTTGTAGGATTCG.

**Lentivirus transduction and siRNA transfection**

Lentivirus production was as described ^[2]^. Cells were exposed to virus-containing supernatants (MOI=5-10) via spinoculation and then sorted by flow cytometry based on GFP or RFP expression. To knockdown CK2, we transiently transfected 293T cells with siCK2 (L-003475-00-0005, Dharmaconn^TM^) or control non-targeting siRNA (D-001810-10-05) using jetPRIME reagent (Polyplus) according to the manufacturer’s protocols.

# Immunoprecipitation (IP) and Western Blotting

Total protein was extracted from cell pellets with RIPA buffer (R0278, Sigma-Aldrich) including Halt phosphatase inhibitor (78420, Thermo Fisher Scientific) and protease inhibitor (78429, Thermo Fisher Scientific) cocktails. Western blotting was as described ^[2]^. Antibodies used were: anti-HMGA1 (Santa Cruz; sc-393213); anti-phos-ser/thr (Cell Signaling Technology; 9631); anti-HMGA1 (N-terminal; GeneTex; GTX100307); anti-phosphoserine (Abcam; ab9332); anti-flag-M2 (Cell Signaling Technology; 2368); anti-SP1 (Santa Cruz; SC-420); and anti-Beta-actin (Santa Cruz; sc-47778).

**Chromatin Immunoprecipitation**

Chromatin immunoprecipitation (ChIP) was performed using the ChIP-IT Express kit (Active Motif) according to the manufacturer’s protocols. 293T cells were transfected with mock vector, HMGA1-WT or HMGA1-S3A, and then crosslinked 10 min with 1% formaldehyde. Cells were washed, lysed and sonicated to obtain 300-500bp fragments. Protein/DNA complexes were incubated with anti-SP1 antibody (santa cruz; SC-420) or normal mouse IgG (santa cruz; sc-2025). Sonicated DNA was amplified using primers for the fragment including reported SP1 binding sites in the BIRC5 promoter (Forward: GACTACAACTCCCGGCACAC; Reverse: GAGCGCACGCCCTCTTAG).

**Cell lines and primary AML cell culture**

Leukemia lines U937, THP1, and MOLM13 were obtained from American Type Culture Collection (ATCC) and cultured in endotoxin-free RPMI1640 supplemented with 10% fetal bovine serum (FBS) (Gemini Bio-Products), 2 mM L-glutamine, 1 mM sodium pyruvate, 100 IU/ml penicillin and 100 μg/ml streptomycin. Mouse MLL-AF9 cells were generated from normal mouse BM progenitor cells (lineage negative, Lin−) transduced with the human MLL-AF9 fusion gene and then maintained in methylcellulose medium for long-term culture. Cell cultures were kept at 37 °C in a humidified incubator with a 5% CO_2_ atmosphere.

**Analysis of cell viability, apoptosis and proliferation**

# Cell growth was measured using a CellTiter-Glo® Luminescent Cell Viability Assay Kit (Promega). Cell cycle parameters were determined using a Click-iT™ EdU Alexa Fluor™ 647 Flow Cytometry Assay Kit (Thermo Fisher). Apoptosis was assessed based on annexin V/DAPI staining (e-bioscience).

**Cell transfection**

293T cells were transfected with various constructs (MOCK/HMGA1-WT/S99A/S102A/S103A/S3A) using JetPRIME reagents (Polyplus) according to the manufacturer’s protocol. All transfections included a GFP vector as transfection control, and successful transduction was indicated by the presence of > 90% GFP-positive cells.

**Quantification and statistical analysis**

Data were analyzed with GraphPad Prism 7 and presented as means ± SEM or means ± SD, as indicated. Two-tailed Student’s t-tests were used to compare means between groups as indicated; *P* < 0.05 was considered significant. Kaplan-Meier survival curves were plotted with GraphPad Prism 7, and *P* values were calculated using the log rank test. For Western blot results, representative figures from three biological replicates were shown. Densitometry analysis of bands from Western blots was performed with a Gel-Pro analyzer and normalized to the loading control.

**Supplementary Table S1-Patient information** (related to Figure 1, Figure 2 and Figure 3).

Demographic features of induction failure or induction remission cohorts used for discovery proteomics and phosphoproteomic analysis. (Therapy: 7+3 IDA+AraC; Abbreviations: IDA, Idarubicin; AraC, Cytarabine. F: Failure; R: Remission; NK: Normal Karyocyte)


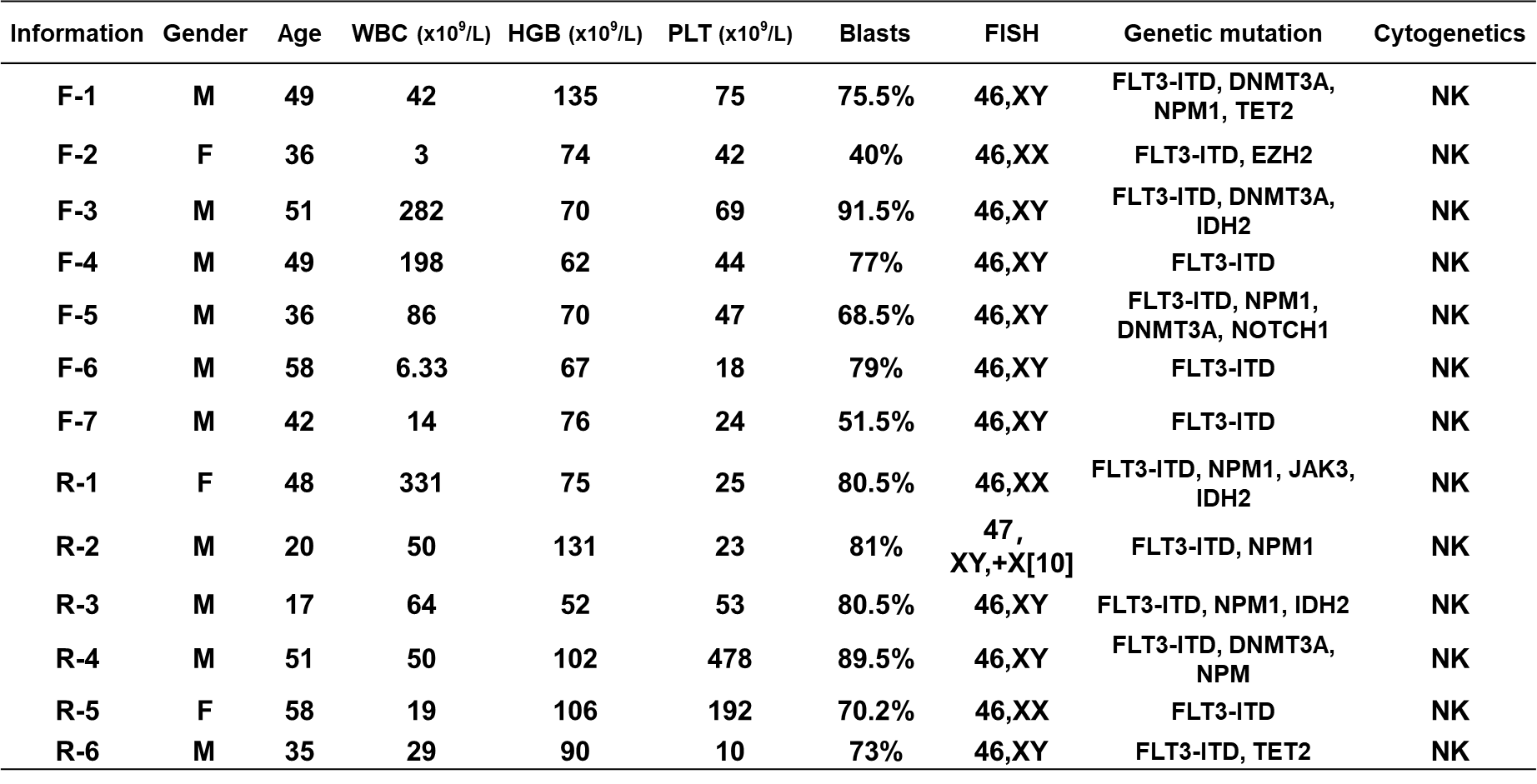


**Supplementary Table S2-Stastical consideration of variation among chemo-failure (F1-F4) and chemo-remission (R1-R4) patients** (related to Figure 1)


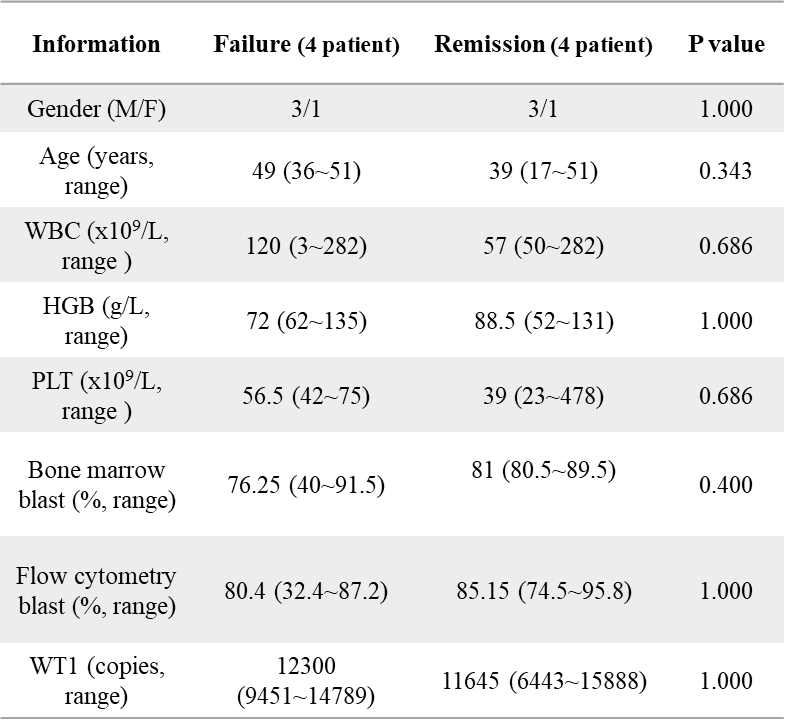


**Supplementary Table S3-Top 24 kinases identified by NetworKIN analysis** (related to Figure 2).


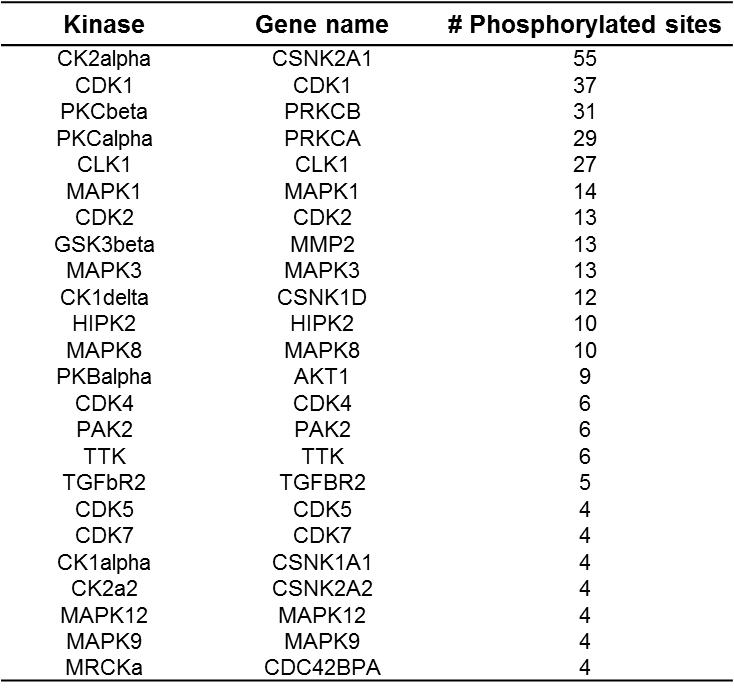


**Supplementary Table S4- KEA2 analysis predicting kinase activity based on presence of differentially phosphorylated proteins** (related to Figure 2).

**
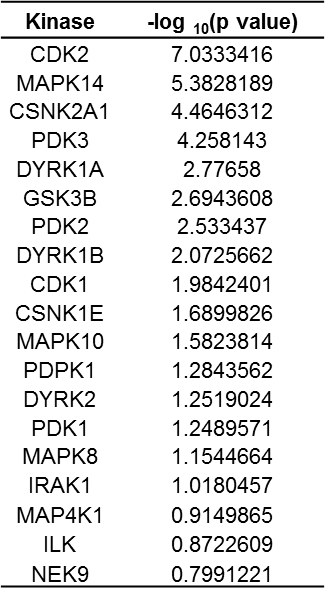
**

**Supplementary Table S5. Proteins shown in 2 IPA terms relevant to hematopoiesis** (related to Figure 3).


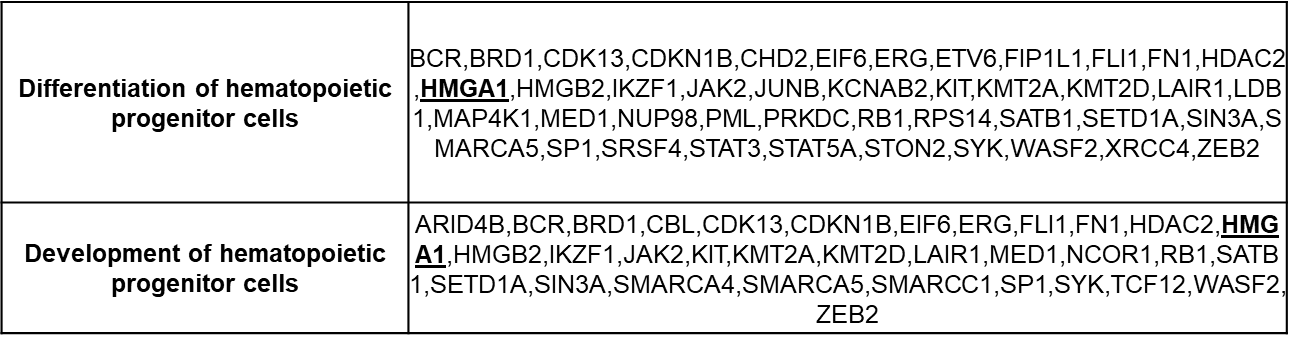


**Supplementary Table S6- Quantification of phosphorylated HMGA1 serines in 8 patients indicated** (related to Figure 3).

**
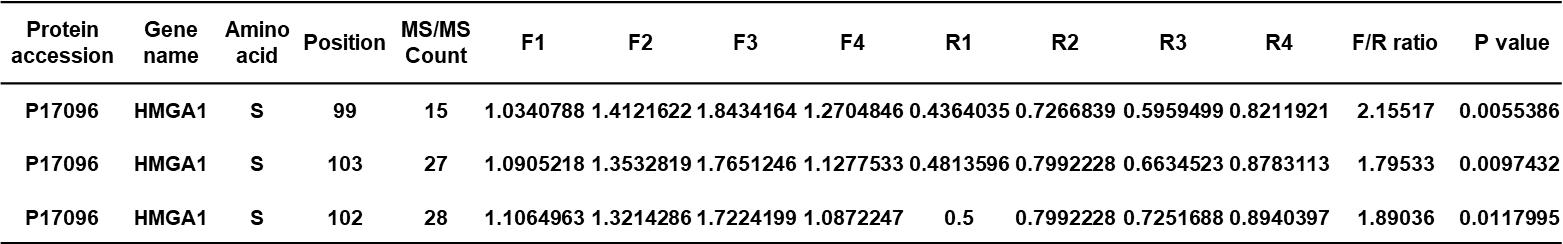
**

**Supplementary Table S7-Detailed information relevant to phosphorylated peptides identified in validation cohort** (related to Figure 3).


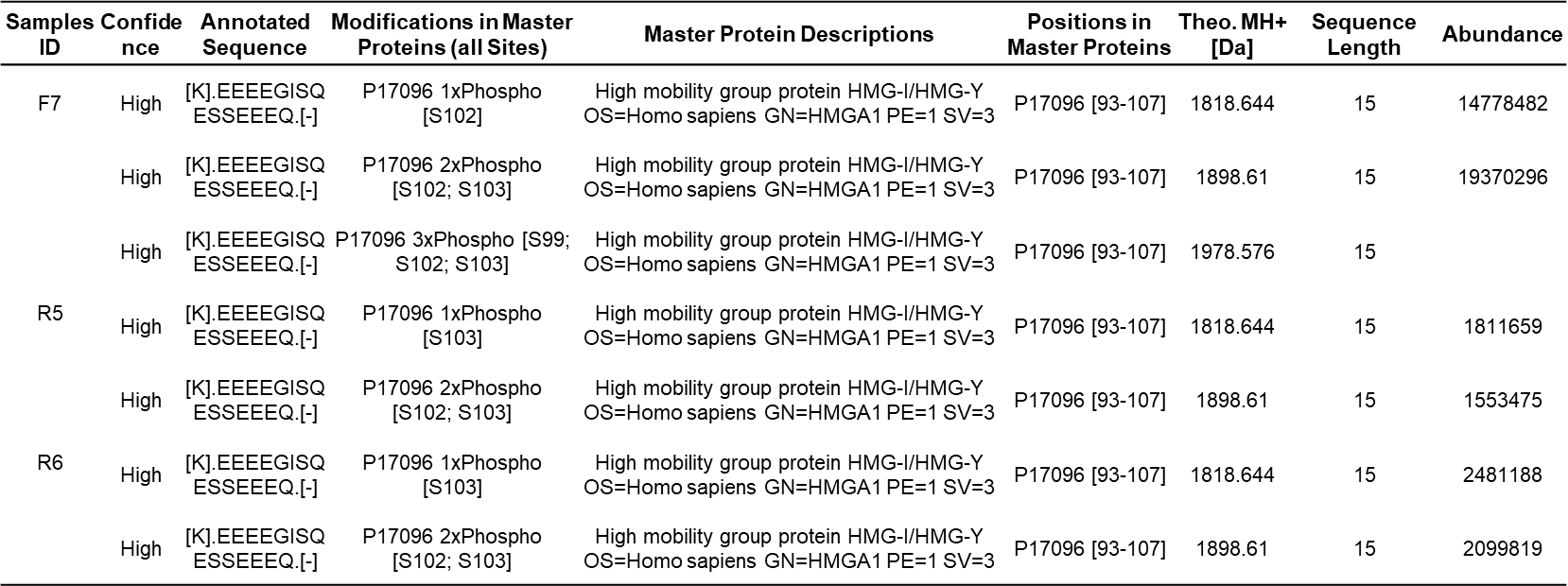

**Figure S1.** (**A**) Mass error and phosphorylated peptide scores indicating that most peptides were identified with high mass accuracy (mass error <1 p.p.m) and a high Andromeda score. (**B, C)** Phospho-peptide length (**B**) and peptide length (**C**) distribution. The length of most (>80%) peptides varied between 7-20 aa, in agreement with properties of tryptic peptides. (**D**) Heatmap showing Pearson’s correlations (R^2^) confirms good overall reproducibility among samples derived from individual patients (F: Failure; R: Remission).

**Figure S2.** (**A**) Altered signaling pathways of IPA gene ontology algorithms for 630 differentially phosphorylated proteins. Pathways are emerged from IPA “Core Analysis”. (**B**) Distribution intensity curves of total phosphorylated proteins and 250 phosphoproteins which are the substrates of 52 corresponding kinases. X-axis: log10-normalized intensities. Y-axis: percentage of protein counts located in each intensity field. (**C**) STRING network showing interaction of enriched kinases in F AML specimens.

**Figure S3**. (**A**) Western blotting for HMGA1 and total phosphor-serine in primary AML cells treated with the CK2 inhibitor CX-4945 (5µM) for 48 hours. Lysate proteins were pulled down using anti-HMGA1 antibody. (**B**) The canonical pathway “Cell death and survival/DNA replication and repair/cell cycle” as analyzed by IPA, was based on differentially phosphorylated proteins. Phosphor-HMGA1 is highlighted.

**Figure S4**. (**A**) Western blotting for HMGA1 and β-actin in indicated cell lines transduced with shCtrl or shHMGA1. (**B-C**) Cell cycle analysis of indicated cells transduced with shCtrl or shHMGA1. (**D**) Western blotting for Flag and β-actin in MA9/ITD cells transduced with Flag-tagged MOCK, HMGA1-S3D or HMGA1-S3A. (**E**) Colony forming cell (CFC) analysis in MA9-ITD cells expressing MOCK, HMGA1-S3D, HMGA1-S99A, HMGA1-S102A, or HMGA1-S103A. 500 cells were plated per well. (**F**) Western blotting of Flag-M2, SP1 and β-actin in 293T cells transduced with MOCK, flag-tagged HMGA1-WT, HMGA1-S3A, HMGA1-T53A. Respective proteins in lysates were pulled down with anti-Flag-M2 antibody. (**G**) Western blotting for Flag and β-actin in 293T cells transduced with Flag-tagged HMGA1-WT or HMGA1-S3A. (**H**) Real-time PCR analysis of SP1 expression in HMGA1-WT- or HMGA1-S3A-expressing 293T cells transiently transduced with siCtrl or siSP1.

References:

1. Zhu, Y., et al., A Comprehensive Proteomics Analysis Reveals a Secretory Path- and Status-Dependent Signature of Exosomes Released from Tumor-Associated Macrophages*.* *J Proteome Res*, 2015. 14(10): p. 4319-31.
2. Sun J, et al. SIRT1 Activation Disrupts Maintenance of Myelodysplastic Syndrome Stem and Progenitor Cells by Restoring TET2 Function. *Cell Stem Cell*. 2018, 23(3):355-369.
